# Distinguishing the mechanisms driving multifaceted plant diversity in subtropical reservoir riparian zones

**DOI:** 10.3389/fpls.2023.1138368

**Published:** 2023-02-24

**Authors:** Jie Zheng, Muhammad Arif, Xinrui He, Xiaolin Liu, Changxiao Li

**Affiliations:** ^1^ Key Laboratory of Eco-environments in the Three Gorges Reservoir Region, Ministry of Education, Chongqing, China; ^2^ Chongqing Key Laboratory of Plant Ecology and Resources Research in the Three Gorges Reservoir Region, School of Life Sciences, Southwest University, Chongqing, China; ^3^ Biological Science Research Center, Academy for Advanced Interdisciplinary Studies, Southwest University, Chongqing, China

**Keywords:** three gorges reservoir, community assembly, environmental filtering, dispersal limitation, plant diversity, biodiversity conservation

## Abstract

Understanding the multifaceted plant diversity and its maintenance mechanisms is crucial for biodiversity conservation. Dam-induced water level fluctuations dramatically alter various aspects of riparian diversity, such as taxonomic (TD), phylogenetic (PD), or functional (FD) diversity. However, few studies simultaneously evaluated plant TD, FD, and PD, especially in the subtropical reservoir riparian zone. Here we sampled plant diversity and environmental drivers along inundation gradients of the Three Gorges Reservoir Region in China. We integrated multifaceted plant diversity to assess how distinct ecological processes affect the plant community assembly and how they respond to inundation gradients, spatial variability, climate, and soils in dam-regulated riparian zones. We found that alpha TD, PD, and FD diversity exhibited decreasing trends with increasing inundation gradients and significant positive correlations with soil organic matter. The number of clustering plant communities increases along the inundation gradients. Beta TD and PD diversity were mainly dominated by species turnover with fewer contributions from nestedness, while beta FD diversity was mainly dominated by nestedness with fewer contributions from species turnover. The explainable rates of different dimensions of beta diversity, turnover, and nestedness ranged from 11% to 61%, with spatial factors explaining the highest beta diversity in different dimensions, followed by inundation gradients, soil properties, and climate variables. Our results suggest dispersal limitations are more important for species turnover in dam-regulated riparian zones at regional scales, while inundation gradients and soil fertility are more critical in shaping plant community assemblages at the local scale. This study emphasizes that environmental and spatial gradients are critical for understanding the assembly mechanisms driving multifaceted plant communities at local and regional scales and reinforces the importance of protecting seed sources and dispersal pathways and maintaining river connectivity when implementing restoration projects.

## 1 Introduction

Riparian zones are critical ecosystems for biodiversity conservation on Earth and offer many ecosystem services to humanity (Rood et al., 2020; Janssen et al., 2022). The riparian plants stabilize the banks and surrounding land surfaces, filter sediment, modulate water nutrient content, and provide habitat for organisms (Gonzalez et al., 2017; Hoppenreijs et al., 2022). Unfortunately, dam construction has caused a catastrophic decline in biodiversity and resulted in the degradation of riparian plants (Wu et al., 2019; Arif et al., 2022). Its adverse effects are global because nearly all large rivers have been dammed in the world, especially in subtropical regions (Azami et al., 2011; Grill et al., 2019). The frequent fluctuations in water flow, especially the shift between inundation and drainage upstream of dams, impose physiological and physical constraints on riparian vegetation (Bejarano et al., 2018), leading to losses in plant species diversity (Zheng et al., 2021b). Furthermore, as plants’ tolerance to this shift is substantially varied, their distribution and dispersion responses to flooding gradients differ (Wang et al., 2021; Ding et al., 2022). However, comprehensive views on how dam-induced water level fluctuations affect riparian plant diversity are still scarce.

Improving biodiversity data is one effective way of addressing the influence of global change on biodiversity, ecological restoration, and species assemblages (GraÇA et al., 2017; Sánchez et al., 2021). Biodiversity in nature is a multi-dimensional property, including taxonomic (TD), phylogenetic (the degree of variety in species lineages; PD), and functional diversity (the degree of variety in species traits; FD) (Faith, 1992; Nooten et al., 2021). These complementary dimensions of biodiversity offer information on ecology and evolution associated with the species assemblages at the α and β diversity scales (Swenson, 2011; Mirochnitchenko et al., 2021). However, most current studies on biodiversity only look at TD, which largely ignores species differences in morphology, physiology, traits, and evolution (Yakimov et al., 2020). Despite the acknowledged importance of the FD and PD for maintaining ecosystem functions and providing ecosystem benefits (Grigoropoulou et al., 2022; Janssen et al., 2022), and recent investigations exploring the filtering of FD by flooding in subtropical reservoirs (Su et al., 2020), there has been insufficient exploration of the impact of flooding on PD in these areas (Dainese et al., 2015). Moreover, studies have shown that dimensions of biodiversity are unequally represented within protected areas and have additionally emphasized the need to apply an integrated perspective to protecting biodiversity (Cadotte and Tucker, 2018; Quan et al., 2018). Therefore, integrating the multi-dimensional framework of TD, PD, and FD to design protection measures for the subtropical reservoir riparian is crucial from a practical standpoint (Doxa et al., 2020). Nevertheless, studies that have simultaneously evaluated plant TD, FD, and PD are scarce (Nakamura et al., 2018; Zheng et al., 2022), and never before in the subtropical reservoir riparian zone. These gaps hinder the implementation of biodiversity conservation strategies in riparian ecosystems due to a lack of comprehensive insight into the assembly mechanisms of multifaceted plants.

Theories of community assemblages, including niche and neutral theory (Sonnier et al., 2014), can be used to address this challenge. The neutral theory holds that under the distinctive roles of ecological drift and distance isolation, stochastic fluctuations (e.g., dispersal filtering) independently determine the patterns of community assembly (Shipley et al., 2012). Plant communities are characterized by poor dispersal and high phylogenetic structure in long-term stable habitats (Grigoropoulou et al., 2022). In contrast, niche theories emphasize the role of environmental filtering in that species could reach one site but are not established because they are unable to endure abiotic environmental constraints (Fraaije et al., 2015). Thus, the community in this habitat is formed by species that have specific functional properties (Giehl et al., 2015). If these properties are conserved in the phylogeny (Yakimov et al., 2020), PD displays low values, and the plant community presents clustering (Grigoropoulou et al., 2022). Meanwhile, Webb et al. (2002) claimed that interspecific competition might result in a community pattern of phylogenetic overdispersion. Numerous studies showed that environmental filtering dominated community assembly under stressful conditions, while dispersal filtering dominated community assembly under benign conditions (He et al., 2022; Wang et al., 2022). However, few investigations have to date studied the extent to which environmental filtering and dispersal limitations drive multifaceted plant diversity at different scales, particularly in the dynamic riparian habitats of the subtropical reservoir.

The elevation in the reservoir riparian zone is often treated as a replacement factor for inundation filters (Janssen et al., 2019; Zhu et al., 2020), as it captures the impact of inundation time on plant diversity patterns (Wang et al., 2021; Ding et al., 2022). Recent investigations showed that plant species diversity peaked at moderate inundation gradients, which can be explained by the moderate disturbance hypothesis (Chen et al., 2020). However, this hypothesis is inadequate to explain this pattern (Fox, 2013). Research shows that the richness patterns may require soil nutrient availability, climate, species dispersal, niche differentiation, and competition to fully explain (Thuiller et al., 2014). In addition, natural seasonal flooding ensures a supply of plant propagules for reservoir riparians, which might enhance available niches and break down the competitive advantage interaction, fundamentally limiting plant community assembly (Merritt et al., 2010). Research showed that propagule dispersal capacity varies across spatial locations in riparian habitats (Beckman et al., 2018). Thus, the mechanisms driving species diversity patterns vary with spatial scale (Giehl et al., 2015), which could be attributed to the differences in climatic and environmental factors between geographical regions (Grigoropoulou et al., 2022). Thus, the plant communities in the riparian zones of reservoirs at different scales might suffer from distinct assembly mechanisms.

In this study, we investigated plant communities and multidimensional variables (soils, climate, and spatial variables) along the inundation gradients within a 30-meter range of hydrological fluctuations within the Three Gorges Reservoir Region (TGRR), China. Here we integrated multifaceted plant diversity (TD, PD, and FD) from different (α and β) scales to evaluate how different ecological processes affect the plant community assembly and how they respond to inundation gradients, spatial variability, climate, and soil in dam-regulated riparian zones. We hypothesized that community assemblages could be expected to shift from overdispersal at high elevations (slight flooding) to clustering at low elevations (severe flooding) due to gradually increasing inundation intensity. For this reason, we expected that TD, PD, and FD would decrease as the inundation gradients increased. However, because of the impact of local environmental filters (e.g., soils) on the species filtered out of the regional species pool by flooding, inundation gradients may not account for all changes in multifaceted diversity. Due to the large geographic gradients examined, spatial factors profoundly influence taxonomic, phylogenetic, and functional β-diversity, which may distinguish from local regional-scale effects of flood disturbance and stress on α-diversity. Specifically, we aimed to answer the following key scientific questions:

(1) What are the patterns and drivers of alpha TD, PD, and FD diversity along inundation gradients in the riparian zone of the TGRR?
(2) What are the patterns and determinants of beta TD, PD, and FD diversity in this region?

## 2 Materials and methods

### 2.1 Study area and vegetation surveying

**Figure 1:**
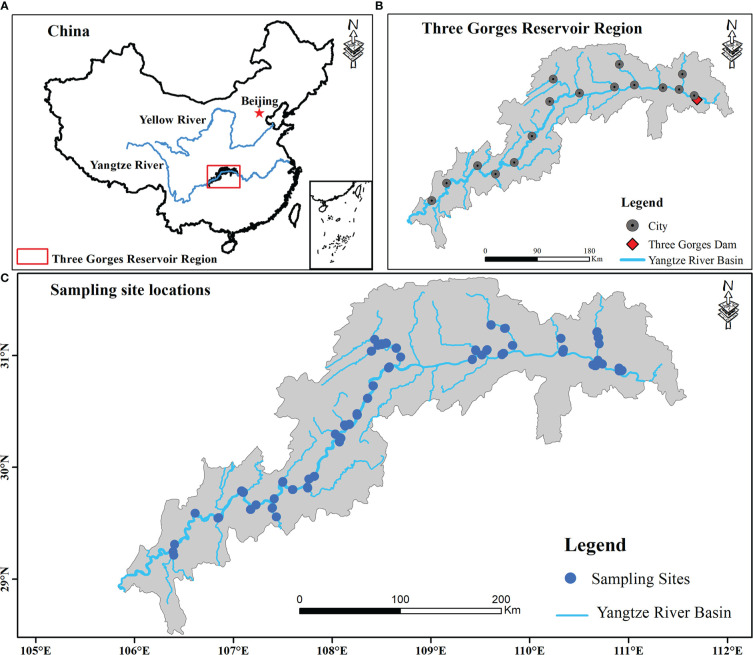
Location of study regions and sampling sites **(B, C)** within the reservoir riparian zone regulated by the Three Gorges Dam on the Yangtze River in China **(A)**.

This study investigated the plant communities along elevational gradients representing different flooding strengths within a freshwater hydro-fluctuation belt of the TGRR in China (a subtropical mega reservoir; **Figure 1**). It includes 16 counties. The climate is mainly influenced by the subtropical monsoon, with average annual temperature (MAT) and precipitation (MAP) being 18.22°C and 1110 mm (Zheng et al., 2021a), of which the primary annual precipitation (about 80%) happens in the rainy season, with daily temperatures ranging from 28°C to 30°C (Yi et al., 2020). Since the first full impoundment of the TGRR in 2010, the periodic inundation and drainage of the TGRR drive large hydraulic fluctuations at elevations of 145-175 m (Ren et al., 2018). The newly formed hydro-fluctuation belt occupies 344.22 km^2^ and covers 639.38 km along the main waterway (Zheng et al., 2021a). The annual human-induced inundation, including rising and falling water levels, lasts for more than eight months, resulting in lower elevations experiencing inundation for longer periods of time (Chen et al., 2020). Under extreme inundation situations, flood-tolerant perennials (e.g., *Cynodon dactylon*, *Hemarthria compressa*) and annual herbs (e.g., *Xanthium strumarium*, *Echinochloa crusgalli*) are dominant in the TGRR (Hu et al., 2022). These plants can still rapidly colonize and establish distinct community types when the inundation recedes, even if their growth is also limited by local environmental factors (e.g., soil).

Under hydrological variations of up to 30 m in the TGRR, lower elevations (145 - 160 m) are subject to longer periods of inundation per year (**Figure S1 and Table S1**), as well as these areas are usually disturbed by natural flooding in summer (Chen et al., 2020). Considering this scenario, the field surveys were conducted in 2019 and 2020 during the period (June-August) when TGRR’s water level reaches its minimum. We collected a total of 327 transects from 36 linked rivers in the riparian zones of the TGRR, encompassing 16 counties within a 58,000 km^2^ landscape (**Figure 1**). Because the plants in our study area have a consistent growing season and are subject to similar inundation disturbances, under these conditions we can use data from two adjacent years for supplementary investigations (Moore et al., 2011; Zheng et al., 2021b). All plant communities from the tail to the dam area were classified according to the differences in water level and assigned to four elevation intervals at 170-175 (zone I), 165-170 (zone II), 160-165 (zone III) and 145-160 m (zone IV), representing 68, 112, 152, and 204 days of inundation continuity, respectively (**Table S1**; Wang et al., 2021). Within each elevation interval, we defined a transect (100 m long) parallel to the river and set up three 2 × 2 m quadrats at an interval of 50 meters (Zheng et al., 2021b). Species presence/absence data were obtained for each elevation interval within 16 counties. In some counties, a sample from four elevation intervals was prevented from being collected simultaneously due to limitations in high water levels (e.g., reservoir tail areas), steep slopes, and landslides (Zheng et al., 2021b). Finally, 981 quadrants were collected in this investigation, including 186, 192, 294, and 309 quadrats in zone I, zone II, zone III, and zone IV, respectively (**Table S1**).

### 2.2 Environmental variables and spatial features

This study investigated soil, topographic, climatic, and spatial parameters closely related to riparian vegetation. First, soils from 0 ~ 20 cm depth in the quadrants were collected to measure 11 soil variables, including soil moisture (SM), pH, bulk density (BD), organic matter (OM), total nitrogen (TN), total potassium (TK), total phosphorus (TP), ammonium ( ${\text{NH}}_{4}^{+}$ -N), nitrate ( ${\text{NO}}_{3}^{-}$ -N), available phosphorus (AP), and available potassium (AK). All soil variables were measured following Bao (1999). Then, the topography of each quadrant was investigated to evaluate the relative elevation, slope, and aspect of each transect. Afterward, MAT and MAP data from January 2000 to January 2018 were calculated using TGRR (http://data.cma.cn/) as a proxy for climatic factors by applying the “extracting data to points” function of ArcGIS10.8. In addition, this study used GPS devices to record the spatial location (latitude and longitude) for all transects. We obtained the spatial variables from the principal coordinates of the neighborhood matrix (PCNM) analysis by applying the “pcnm” procedure in the package “vegan” (Borcard and Legendre, 2002; Oksanen et al., 2020).

### 2.3 Plant trait and phylogenetic data

This study selected five key traits related to aspects of the ecological strategies of riparian plants and provided information on functional characteristics that environmental filters could potentially select (Brice et al., 2017). These functional traits include dispersal type, growth form, life cycle, shoot height, and flowering phenology (Yi et al., 2020; **Table S2**). These traits have proven to be ideal indicators for revealing plant functional characteristics in the TGRR (Yi et al., 2020). This research established a list of 166 species belonging to 43 families (**Table S3**). We then used the latest mega-phylogeny of seed plants as a backbone to construct species-level phylogenetic trees using the “V.PhyloMaker” package, which comprises 74,533 vascular plants (Smith and Brown, 2018; Jin and Qian, 2019), comprising 166 species from the present investigation.

### 2.4 Multidimensional plant diversity

Alpha TD and FD of plant communities were measured by applying the Rao quadratic entropy (RaoQ) index, which reflects the relative abundance and diversity of species (Rao, 1982).


$$RaoQ=\sum_{i=1}^{s}\sum_{j=1}^{s}d_{ij}p_{i}p_{j}$$

where *d*_ij_ is the functional distance between species *i* and *j* (d*ij* = 0 for *i* = *j*), and *p*_i_ and *p*_j_ are the relative abundances of the *i*th and *j*th species. The RaoQ index was computed by applying the “ade4” package (Doxa et al., 2020). In this analysis, the Gower distance of species traits was computed by applying the “FD” package. In addition, this study used Faith’s phylogenetic diversity, mean pairwise distance (MPD), and mean nearest taxon distance (MNTD) to represent alpha PD (Faith, 1992; Qian et al., 2019). Here we use MPD and MNTD because they exhibit comparatively lower type I errors and allow for investigating community structure variations at deeper and shallower phylogenetic levels (Miller et al., 2017). To infer the mechanisms of plant community assembly, the standardized effect sizes (SES) of PD and FD diversity were computed by a null model (Luo et al., 2019). SES values were estimated according to the following equation.


$$SES=\frac{Div_{obs}-Div_{null}}{sd_{null}}$$

where Div*_obs_* is the observed diversity of plant communities, Div*_null_* is a random community’s expected mean diversity, and sd*_null_* is the standard deviation of 1000 Div*_null_* values. This process maintains the observed species richness, with SES > 0 indicating overdispersal and SES< 0 indicating clustering. Significant deviations from random are indicated if SES > 1.96 or SES< - 1.96 (Webb et al., 2002; Yakimov et al., 2020).

To quantify beta diversity, presence/absence data, species functional distances (characterized by Gower distances for five species traits), and phylogenetic trees were employed to generate beta TD, FD, and PD diversity, respectively. In addition, the overall beta diversity was decomposed into turnover and nestedness components by using the package “betapart” (Baselga, 2010; Baselga and Orme, 2012). In this package, the “beta.pair”, “phylo.beta.pair” and “func.beta.pair” procedures were employed to generate overall beta TD, PD, and FD diversity, respectively. The “beta.multi”, “phylo.beta.multi” and “func.beta.multi” procedures were employed to generate multifaceted turnover and nestedness components (Baselga and Orme, 2012).

### 2.5 Statistical methods

This study employed linear mixed models (LMMs) to investigate the influence of soil, topographic, and climatic parameters on alpha TD, FD, and PD diversity. Thirty-six selected rivers were included as nested random effects (intercepts). Because soil and climate parameters were strongly correlated, principal component analysis was used for climate and soil variables before parameter estimation for a limited number of variables. The PCA axes with an interpretation rate higher than 10% were retained for subsequent analysis (Slik et al., 2013; Zheng et al., 2022), resulting in the top axis of the climatic parameter (Climate_PC1) as well as the top four axes of soil parameters. Climate_PC1 is correlated with temperature and precipitation. The first four PCA axes of the soil variables were linked to soil phosphorus (Soil_PC1), nitrogen (Soil_PC2), water content (Soil_PC3), and organic matter (Soil_PC4), respectively. Then, all predictors were centered and scaled by their means and SD to make them comparable for interpreting direction and magnitude in all LMMs (Luo et al., 2019). All combinations of fixed effects were examined and the optimal model was identified based on the Akaike Information Criterion (AIC) (Maestre et al., 2012). This analysis was performed in the “nlme” package, whose “corSpher” procedure was applied to calculate the spatial autocorrelation among transects in this study (Maestre et al., 2012).

In addition, we investigated the correlation between multidimensional beta diversity with spatial and environmental distance by employing the “ecodist” package (Goslee and Urban, 2007). Finally, we applied variance partitioning to uncover the relative contributions of environmental and dispersal filtering in driving beta diversity patterns (Zheng et al., 2021b). Redundancy analysis and forward selection were executed to reduce redundant components in this analysis (Zheng et al., 2021a). The ‘vegan’ package partitions the total explained variance (*R* ^2^) into three parts, including their unexplained, joint, and independent effects (Oksanen et al., 2020). Given the difference in the number of explanatory factors for this model, the adapted *R* ^2^ was employed to represent the explanatory strength for the different components (Zheng et al., 2022).

## 3 Results

### 3.1 Multifaceted plant alpha diversity along the inundation gradients and response to environmental factors

**Figure 2:**
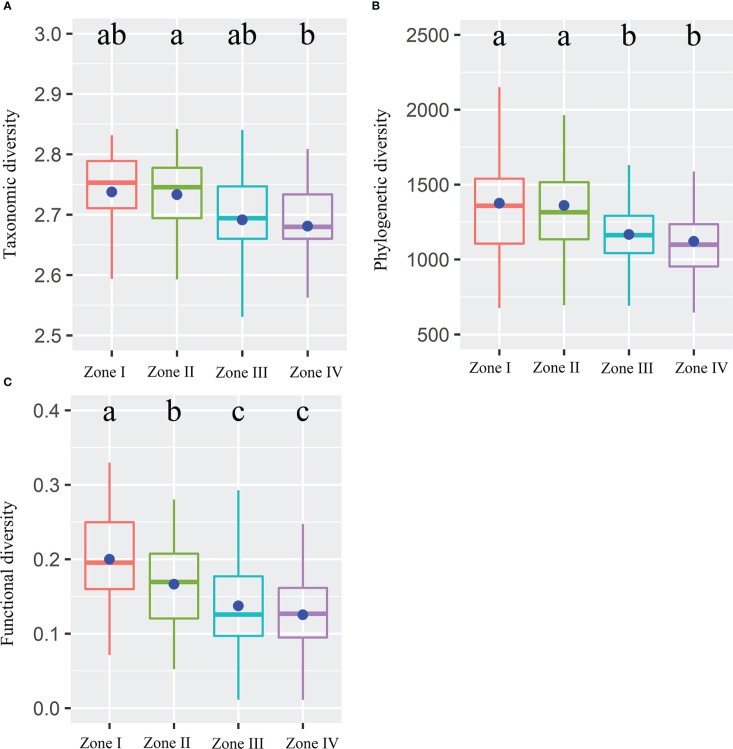
Taxonomic **(A)**, phylogenetic **(B)**, and functional **(C)** diversity within four inundation gradients within the reservoir riparian zone regulated by the Three Gorges Dam in China. Boxes present the median, 25th, and 75th percentile. The blue dots of boxes represent the average values. Different letters are used to mark significantly distinct values between inundation gradients based on the Kruskal-Wallis test (p < 0.05).

**Figure 3:**
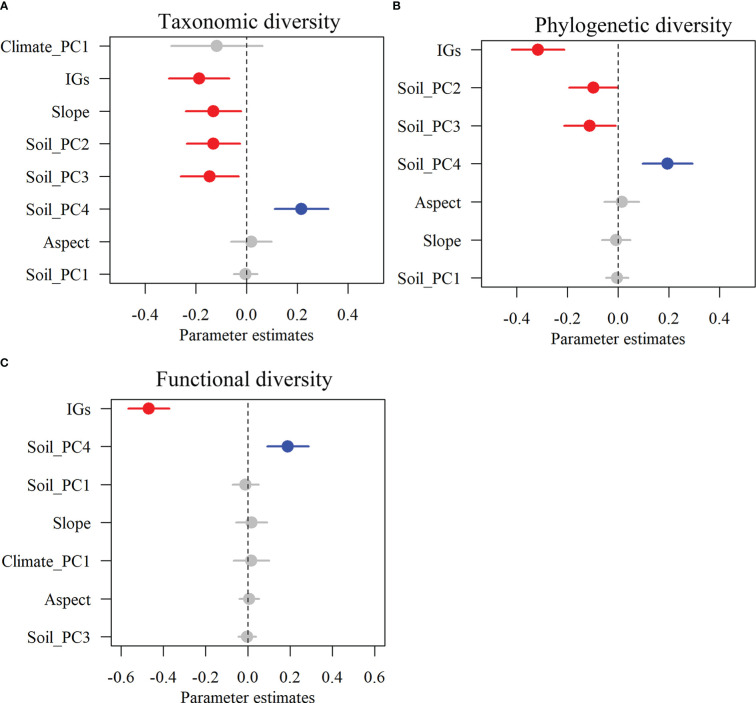
The effects of multi-dimensional variables on multifaceted plant alpha diversity. Taxonomic diversity **(A)**, phylogenetic diversity **(B)**, and functional diversity **(C)**. Dots with error bars denote the effect sizes and 95% confidence interval (CI), respectively. The blue and red colors represent significant positive and negative effects, respectively. Climate_PC1 (climate), Soil_PC1 (soil phosphorus), Soil_PC2 (soil nitrogen), Soil_PC3 (soil water content), Soil_PC4 (soil organic matter), Slope (slope), Aspect (aspect), and IGs (inundation gradients).

Alpha TD, PD, and FD indices showed a gradual decrease with increasing inundation intensity (**Figure 2**). TD values in zone II were remarkably greater than those in zone IV. Still, the differences were insignificant in the rest of the cases (**Figure 2A**). The PD values in zones I and II were remarkably greater than those in zones III and IV. Still, there were no significant differences between zones I and II and between zones III and IV (**Figure 2B**). The FD values were remarkably greater in Zone I than in all other zones and in Zone II than in Zones III and IV. Still, there was no significant variation between Zones III and IV (**Figure 2C**). The results showed that TD showed significant negative correlations with IGs, Slope, Soil_PC2, and Soil_PC3. In contrast, it showed significant positive correlations with Soil_PC4 (**Figure 3A**). PD showed significant negative correlations with IGs, Soil_PC2, and Soil_PC3. In contrast, it showed significant positive correlations with Soil_PC4, showing a significant positive correlation (**Figure 3B**). PD showed significant negative and positive correlations with IGs and Soil_PC4, respectively (**Figure 3C**). Overall, inundation intensity and soil organic matter showed significant negative and positive effects on alpha TD, PD, and FD diversity, respectively.

**Figure 4:**
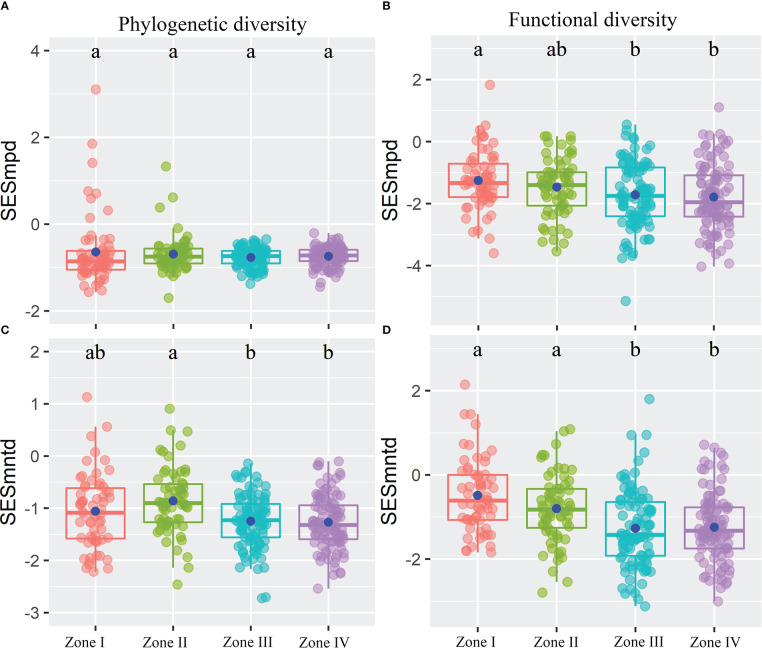
The standardized phylogenetic **(A, C)** and functional **(B, D)** structures of plant communities within the reservoir riparian zone regulated by the Three Gorges Dam in China. Boxes present the median, 25th, and 75th percentile. The blue dots of boxes represent the average values. Different letters are used to mark significantly distinct values between inundation gradients based on the Kruskal-Wallis test (p < 0.05).

**Figure 5:**
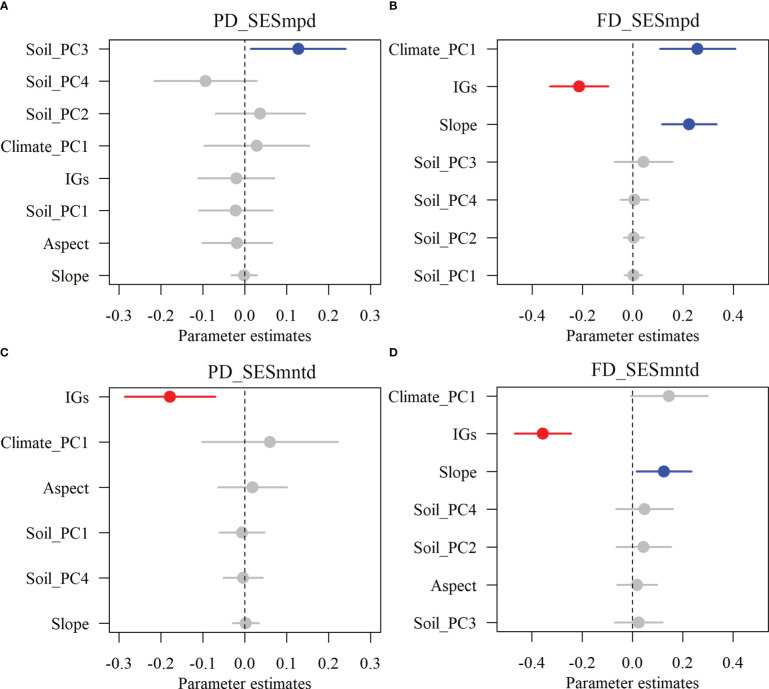
The effects of multi-dimensional variables on standardized phylogenetic **(A, C)** and functional **(B, D)** structures of plant communities. Dots with error bars denote the effect sizes and 95% CI, respectively. Blue and red colors represent significant positive and negative effects, respectively. Climate_PC1 (climate), Soil_PC1 (soil phosphorus), Soil_PC2 (soil nitrogen), Soil_PC3 (soil water content), Soil_PC4 (soil organic matter), Slope (slope), Aspect (aspect), and IGs (inundation gradients).

The results showed that inundation intensity did not significantly affect the SESmpd index for PD. In contrast, they significantly affected both SESmntd for PD and SESmpd and SESmntd for FD (**Figure 4**). Only one community with SESmpd of PD and SESmntd of FD values higher than 1.96 was in an overdispersion pattern in light inundation intensity. Most plant communities were in a clustering pattern (standardized values less than zero), and the number of clustering plant communities increased gradually with increasing inundation intensity (**Figure 4**). Furthermore, SESmpd of PD exhibited a significant positive relationship with Soil_PC3 (**Figure 5A**). SESmntd of PD exhibited a significant negative relationship with IGs (**Figure 5C**). SESmpd of FD exhibited significant positive relationships with Soil_PC1 and slope, while it showed significant negative correlations with IGs (**Figure 5B**). SESmntd of FD exhibited significant negative and positive relationships with IGs and slope, respectively (**Figure 5D**).

### 3.2 Multifaceted plant beta diversity and their ecological drivers

**Figure 6:**
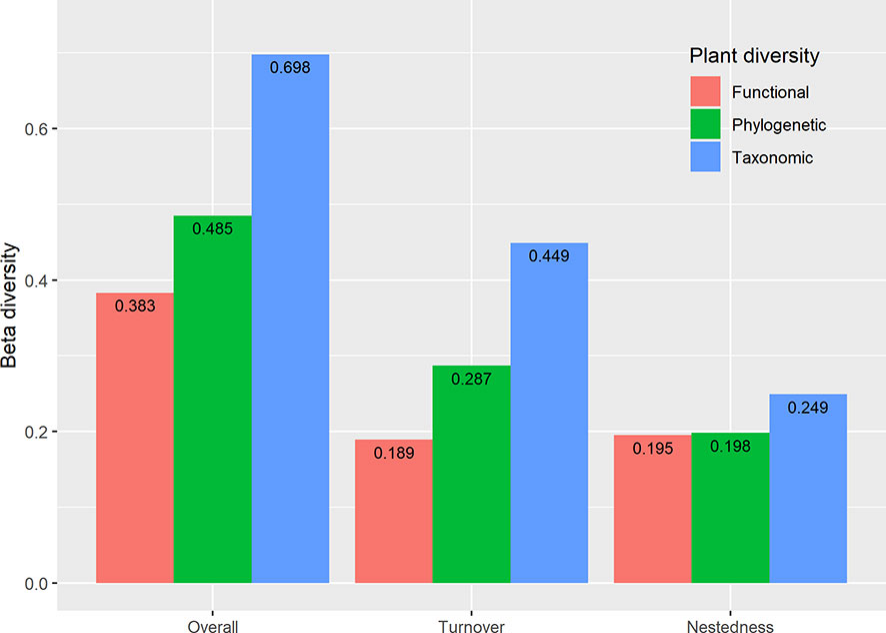
Multifaceted plant overall beta diversity, turnover, and nestedness within the reservoir riparian zone regulated by the Three Gorges Dam on the Yangtze River in China.

The results showed extensive variation in taxonomic overall beta diversity (0.698) paired measures (**Figure 6**). Still, relatively little variation in functional (0.383) and phylogenetic (0.485) overall beta diversity (**Figure 6**). Beta TD and PD diversity were dominated by species turnover (64.33% and 59.18%, respectively), but the contribution of nestedness was relatively low (35.67% and 40.82%, respectively). By contrast, nestedness (50.77%) has a larger contribution to beta FD diversity than turnover (49.23%).

The results showed a high rate of species turnover between plant communities along inundation gradients (**Table 1**). After considering the effects of spatial variables, environmental variables showed significant positive relationships with beta diversity and its components in different dimensions, except for taxonomic nestedness (**Table 1**). After considering the effects of environmental variables, spatial variables showed significant positive correlations with beta diversity and its components in different dimensions, except for phylogenetic and functional nestedness (**Table 1**).

**Table 1:** Mantel correlations between multifaceted plant beta diversity and environmental and spatial factors.

Variables	Tax.sor	Tax.sim	Tax.sne	Phy.sor	Phy.sim	Phy.sne	Fun.sor	Fun.sim	Fun.sne
Environment	0.22**	0.16**	0.03ns	0.15**	0.08**	0.07*	0.16**	0.09**	0.07*
Spatial	0.31**	0.29**	0.06*	0.22**	0.19**	0.02ns	0.21**	0.22**	0.01ns

Tax.sor, Tax.sim, and Tax.sne represent taxonomic beta diversity, turnover, and nestedness, respectively. Phy.sor, Phy.sim, and Phy.sne represent phylogenetic beta diversity, turnover, and nestedness, respectively. Fun.sor, Fun.sim, and Fun.sne represent functional beta diversity, turnover, and nestedness, respectively. The ns represents no significant difference (P > 0.05), *P< 0.05, **P< 0.01.

**Figure 7:**
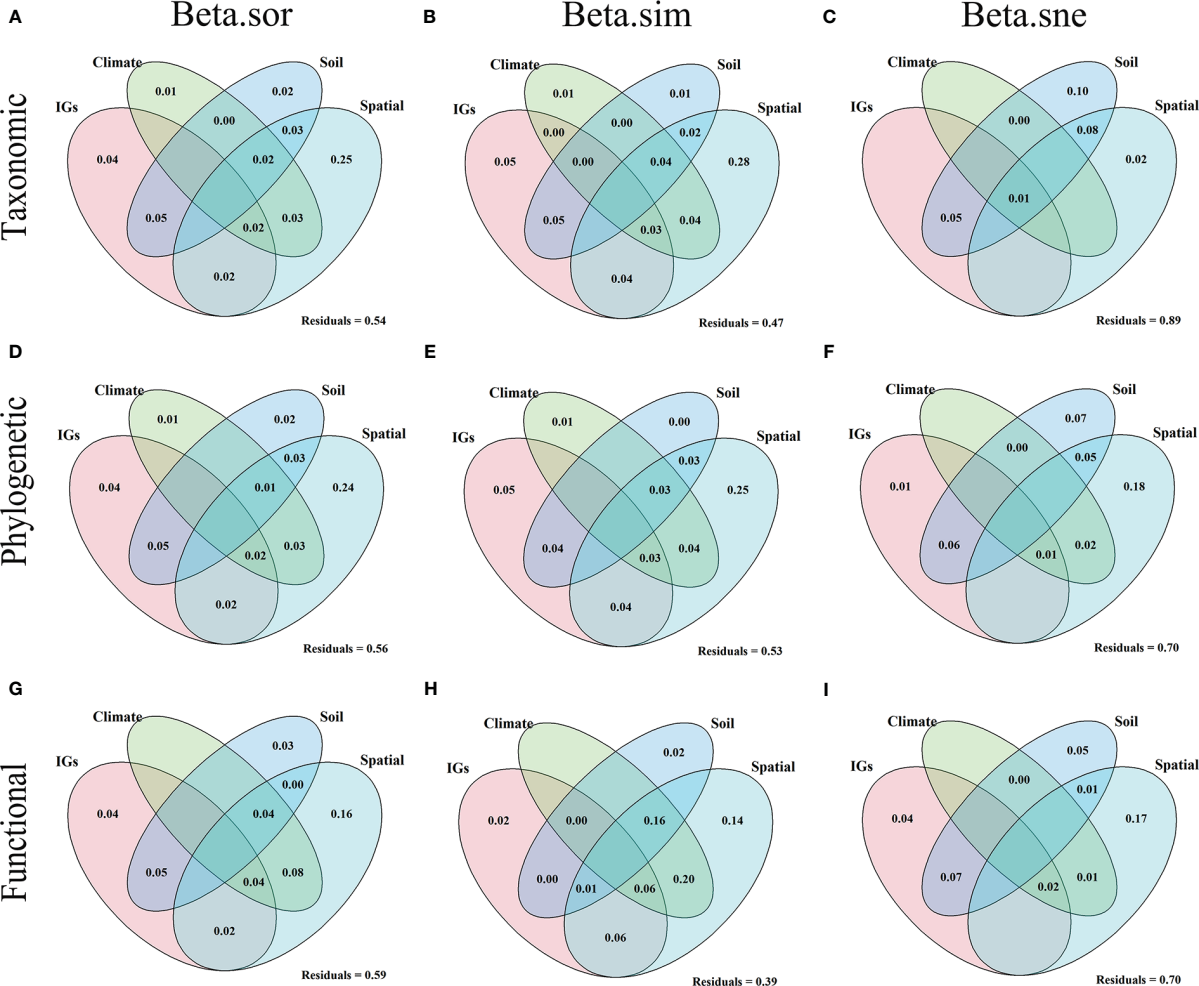
Venn diagram based on variance partitioning reveals the variation in taxonomic **(A–C)**, phylogenetic **(D–F)**, and functional **(G–I)** beta diversity explained independently and jointly by inundation gradients (IGs), climate, soil, and spatial factors. The variance (adjusted R2) was explained by inundation gradient (elevation), climate (average annual temperature and precipitation), soil (all soil indicators measured), and spatial factors (all PCMN vectors).

Results showed explainability of β-diversity and its components varied from 11% to 61% (**Figure 7**). For the β-diversity component of TD (**Figures 7A–C**), among the pure effects, spatial factors explained the entire β-diversity (25%), turnover (28%), and nestedness (2%). Soil factors explained the entire β-diversity (2%), turnover (1%), and nestedness (1%). Climatic factors explained the entire β-diversity (1%) and turnover (1%). Inundation intensity (IGs) explained the entire β-diversity (4%) and turnover (5%). For the β-diversity component of PD (**Figures 7D–F**), in the pure effect, spatial factors explained the entire β-diversity (24%), turnover (25%), and nestedness (18%). Soil factors explained the entire β-diversity (2%) and nestedness (7%). Climatic factors explained the entire β-diversity (1%) and turnover (1%). Inundation intensity explained the entire β-diversity (4%), turnover (5%), and nestedness (1%). For the β-diversity component of FD (**Figures 7G–I**), in pure effects, spatial factors explained entire β-diversity (16%), turnover (14%), and nestedness (17%). Soil factors explained entire β-diversity (3%), turnover (2%), and nestedness (5%). Inundation intensity explained entire β-diversity (4%), turnover (2%), and nestedness (4%). Overall, the pure effects of the spatial factors explained the highest amount of the total variance, followed by inundation intensity, soil, and climate. These variables explained more variation in the entire β-diversity and turnover in different dimensions than in the nestedness.

## 4 Discussion

### 4.1 Environmental filtering dominates plant species assemblage in dam-regulated riparian zones

Although alpha and beta scale approaches in different dimensions have been relatively common in various ecosystems (Arnan et al., 2017; Hill et al., 2019), there are no comparable concerns about plant communities in dam-regulated riparian zones. In this study, TD, PD, and FD diversity across different scales (α and β) were integrated for the first time to fully understand the influence of dam impoundment on plant assemblages of subtropical reservoir riparian zones. Our study showed that alpha TD, PD, and FD diversity decreased gradually with increasing inundation gradients and was significantly influenced by inundation intensity and soil organic matter (**Figures 2**, **3**), suggesting that inundation intensity and soil fertility drive the plant diversity patterns of TD, PD, and FD along inundation gradients of reservoir riparian zones. Previous studies found that the inundation depth, inundation frequency, and inundation duration significantly affected plant growth (Chen et al., 2020; Zhang and Xie, 2021) and that species diversity was significantly higher at higher elevations than at lower elevations in the reservoir riparian zones (Zheng et al., 2021b). Inundation stress can directly filter out riparian plants that lack resistance to flood stress or do not recover quickly from flood stress (Bejarano et al., 2020). Inundation stress can also indirectly alter plant community abundance by changing soil properties (e.g., soil fertility) (Giehl et al., 2015). Under fluctuating reservoir hydrology, there may be an enrichment of soil nutrients at lower elevations of the drawdown zone of the reservoir (Ran et al., 2022). This situation can increase the nutrient supply capacity of these areas, resulting in a decrease in FD with increased nutrient availability (Mugnai et al., 2022). In the hydro-fluctuation belt of the TGRR, soil nutrients show an increasing trend with decreasing elevation (Ran et al., 2022), which may be one of the reasons for the lower FD diversity at lower elevations. Conversely, functional traits are distinct in infertile soils, and high functional richness occurs under this condition (Giehl et al., 2015). Research has revealed that plant functional traits mostly converge throughout phylogeny after a long period of inundation (Giehl et al., 2015). Plant PD and FD diversity should usually show similar patterns when functional plant traits exhibit clear phylogenetic signals (Bello et al., 2017). Furthermore, it has also been suggested that although hydrological stress is important for plant diversity, it does not fully explain diversity patterns in reservoir riparian zones (Giehl et al., 2015). Soil fertility is also an important variable in shaping plant community assembly.

Under the influence of hydrological changes in the TGRR, most plant communities had negative values of standardized PD and FD diversity (**Figure 4**), suggesting that environmental filtering dominates plant assemblages in dam-regulated riparian zones (Webb et al., 2002). A powerful explanation is that frequent shifts in reservoir hydrology inhibit the recruitment process of riparian plant communities (Yi et al., 2019). These useful findings are consistent with previously reported conclusions that plant species assemblage along a hydrologic gradient is shaped by environmental filtering (Fraaije et al., 2015; Ran et al., 2022). Furthermore, Ran et al. (2022) highlighted that the competitiveness among plants in the reservoir riparian zones may be insufficient for separating species niches, and this potentially weakens other community assembly processes. It is worth mentioning that the present study still detected positive values of standardized PD and FD diversity in some plant communities, especially in the higher elevation zones of the reservoir riparian zones (**Figure 4**). This result implies that those plant communities in these zones are overdispersed, which may be due to interspecific competition. Studies showed that interspecific competition in a benign environment may be important for community assembly (Luo et al., 2019). Among the dominant plants in the TGRR, *Cynodon dactylon* clones have regenerative abilities (Zhu et al., 2020). This characteristic can help them recover quickly after being subjected to periodic flooding, thus improving their competitiveness with other species (Zhang and Xie, 2021). Overall, these studies support our finding that plant species assemblage in reservoir riparian zones is dominated by environmental filtering.

### 4.2 Patterns of multifaceted plant beta diversity in reservoir riparian zones

Our result confirms that beta TD and PD diversity prevailed by turnover, while beta FD diversity prevailed by nestedness (**Figure 6**), suggesting spatial mismatches between beta TD, PD, and FD diversity (Doxa et al., 2020). Habitat heterogeneity is often considered important in promoting turnover rates (Ahmad et al., 2020). Our study areas are large (344.22 km^2^), span 639.38 km vertically, and have a pronounced physical gradient (Zheng et al., 2021a). This study area has variable geo-climatic conditions, and agricultural activities have profoundly altered the overall environmental conditions of the watershed (Arif et al., 2020). In addition, differences in non-native species invasions may lead to a further reduced proportion of spatially shared species riparian zones (Brice et al., 2017), resulting in increased species turnover. Studies have reported that exotic plants in the TGRR are rapidly increasing and significantly altering the plant community species composition (Wang et al., 2021; Zhang and Xie, 2021; Hu et al., 2022). Therefore, these factors probably contribute to the high rate of plant species turnover in the TGRR (Coelho et al., 2018).

Our study also found higher rates of taxonomic turnover than phylogenetic turnover (**Figure 6**), suggesting that recent lineage diversity generates distinct but closely related plant communities (Valente et al., 2010). Studies suggested that recent lineage diversity is one driver for higher taxonomic turnover rates than phylogenetic turnover rates (Valente et al., 2010). Moreover, Swenson (2011) highlighted that phylogenetic turnover rates are less than taxonomic turnover rates because of sharing branch lengths among species. Therefore, maintaining habitat heterogeneity is essential to improving plants’ taxonomic and phylogenetic diversity in the TGRR. Furthermore, our results suggest that nestedness dominates functional beta diversity (**Figure 6**), which may result from strong fluctuations in extreme environments or the short term, possibly through selective extinction (environmental filtering). Species with multiple differences in traits are found in places that feature diverse habitat types, while only those species with certain traits are favored in a homogeneous habitat. Previous studies have found that riparian plant functional traits tend to exhibit convergent evolution under intense hydrological stress (Giehl et al., 2015; Brice et al., 2017). Therefore, the periodic inundation conditions of the TGRR may have led to the homogenization of riparian plant traits. Furthermore, Perez Rocha et al. (2019) suggested that the predominance of nesting in functional beta diversity may also be because both trait composition and habitat requirements vary in nested forms. Thus, lower functional turnover and higher functional nesting suggest that more homogeneous plant functional traits may have developed in the TGRR.

### 4.3 Ecological drivers of beta diversity

Our findings suggest that the interpretable rates of beta TD, PD, and FD diversity and its components ranged from 11% to 61%, with spatial factors explaining the highest amount of beta diversity in different dimensions, followed by inundation intensity, soils, and climate variables (**Figure 7**). These results suggest that plant beta diversity in different dimensions is mainly shaped by spatial factors through dispersal limitation. Recent studies suggest that spatial variables may be associated with dispersion-related mechanisms (ie, dispersal filtering) (Grigoropoulou et al., 2022). Larger spatial distances pose a challenge to species dispersal and perform critical functions for determining plant community assemblages within wide spatial areas (Mugnai et al., 2022). In our study region, the velocity of water flow is affected by reservoir impoundment, especially near the upstream of the dam, where the reservoir level remains almost constant (Zheng et al., 2021a). Studies showed that the water flow velocity in the TGRR decreases 20 times after the Three Gorges Dam impoundment (Su et al., 2013), which would pose a considerable dispersal barrier for many plants (Merritt et al., 2010). Thus, spatial factors may have largely driven the changes in plant beta diversity in the riparian zones of the TGRR. In addition, a large amount of variation in beta diversity remains unexplained because our model is unable to simulate stochastic events (ie, species colonization and extinction) and interactions among species within the community. Indeed, reservoir riparian zones are always subject to continuous and variable external disturbances, which may lead to stochastic extinction and recolonization of plant species. Therefore, it is difficult to comprehensively predict variations in the composition of plant communities in dam-regulated riparian zones.

### 4.4 Implications for biodiversity conservation in the reservoir riparian zones

Effective biodiversity protection efforts should consider the maintenance of species, functions, and evolutionary processes at local and regional scales (Cadotte and Tucker, 2018). Knowledge of beta diversity patterns can provide important evidence for predicting ecosystem function and improving protected priorities (Bergamin et al., 2017). For example, if species turnover is the dominant pattern, more protected areas are needed to conserve biodiversity, and when nesting is the dominant pattern, a sufficiently large and species-rich protected area needs to be established (Bergamin et al., 2017). For the plant communities of the TGRR, multi-faceted beta diversity is characterized by different components (**Figure 6**), so policymakers need to implement various conservation strategies with different conservation objectives and targets (Cadotte and Tucker, 2018). From a taxonomic and phylogenetic perspective, it is more appropriate to conserve multiple habitats to protect ecological communities of different strains as turnover rates dominate. However, from a functional perspective, due to the predominance of nesting, the conservation priorities should be larger nature reserves, and regions that possess greater diversity need to take priority for conservation to maintain the functional integrity of the regional ecosystem.

In addition, this study provides guidelines for vegetation restoration and management in the reservoir riparian zones. Since both environmental and spatial variables have significant contributions in influencing beta diversity (**Table 1** and **Figure 7**), combining environmental filtering with spatial factors (diffusion limitation) is advisable. For the reservoir riparian zones, a reasonable configuration of plants with different adaptations based on the difference in flooding intensity at different elevations is needed to restore effective riparian vegetation (Ding et al., 2022). In addition, proactive restoration measures are required to address barriers to seed dispersal and seedling development. Therefore, the protection of seed sources and their dispersal pathways is necessary. For example, as plant propagules spread through water, which is a critical driver of riverine plant diversity, maintaining river connectivity can effectively conserve riparian plant diversity. Considering that the construction of dams will likely continue to increase as global flood control, power generation, navigation and diversion, and the demand for clean energy increase, this implies a greater threat to riparian habitats. Therefore, our results are of great practical value in guiding the phytoremediation and management of degraded riparian ecosystems and biodiversity conservation.

## 5 Conclusion

In summary, this study integrates taxonomic diversity, phylogenetic diversity, functional diversity, and environmental drivers to evaluate general plant patterns and assembly mechanisms along an inundation gradient across the TGRR hydro-fluctuation belt. Our study demonstrated that inundation intensity and soil fertility drive the patterns of multifaceted plant diversity along inundation gradients of the TGRR. Only a few plant communities are overdispersed in high-elevation zones, while most of them are clustered in all elevation zones. The number of clustering plant communities gradually increases as the elevation decreases, indicating that environmental filtering dominates the plant species assemblage in dam-regulated riparian zones. In addition, beta taxonomic and phylogenetic diversity was primarily driven by turnover, with less contribution from nestedness. In comparison, functional beta diversity was mainly dominated by nested components with less contribution from turnover components. This suggests that the patterns of multifaceted beta diversity are distinct in the riparian zones of the TGRR. Thus, the conservation of plant diversity should depend on specific needs. Furthermore, the interpretable rates of multifaceted plant beta diversity and its components ranged from 11% to 61%, with spatial factors explaining the highest amount of beta diversity in different dimensions, followed by inundation intensity, soil, and climate variables. These results suggest that spatial factors more strongly determine plant beta diversity in different dimensions through dispersal limitation. Overall, our results emphasize that conserving riparian plant diversity requires protecting seed sources and dispersal pathways and maintaining river connectivity and reinforces the importance of incorporating multiple biodiversity dimensions for biodiversity conservation in subtropical reservoir riparian zones.

## Data availability statement

The original contributions presented in the study are included in the article/**Supplementary Material**, further inquiries can be directed to the corresponding author.

## Author contributions

Conceptualization, Methodology, Software, Investigation, Visualization, Writing - original draft, Writing - review & editing were performed by JZ. Formal analysis, Writing - review & editing were performed by MA. Investigation and Formal analysis were performed by XH and XL. Writing - review & editing, Supervision, Project administration, and Funding acquisition were performed by CL. All authors contributed to the article and approved the submitted version.
