# Supplementary material for: Distinguishing the mechanisms driving multifaceted plant diversity in subtropical reservoir riparian zones
Source: Front Plant Sci. 2023 Feb 24;14:1138368. doi: 10.3389/fpls.2023.1138368 (PMC9998900; doi:10.3389/fpls.2023.1138368)
Supplement: Supplementary file 1 [file DataSheet_1.docx]

***Supplementary Material***

**Distinguishing the mechanisms driving multifaceted plant diversity in subtropical reservoir riparian zones**

**Jie Zheng ^1^, Muhammad Arif ^1,2^, Xinrui He ^1^, Xiaolin Liu ^1^, Changxiao Li ^1,2,^ ***

*** Correspondence:** Corresponding Author: Changxiao Li, lichangx@swu.edu.cn

# 1. Supplementary Figures


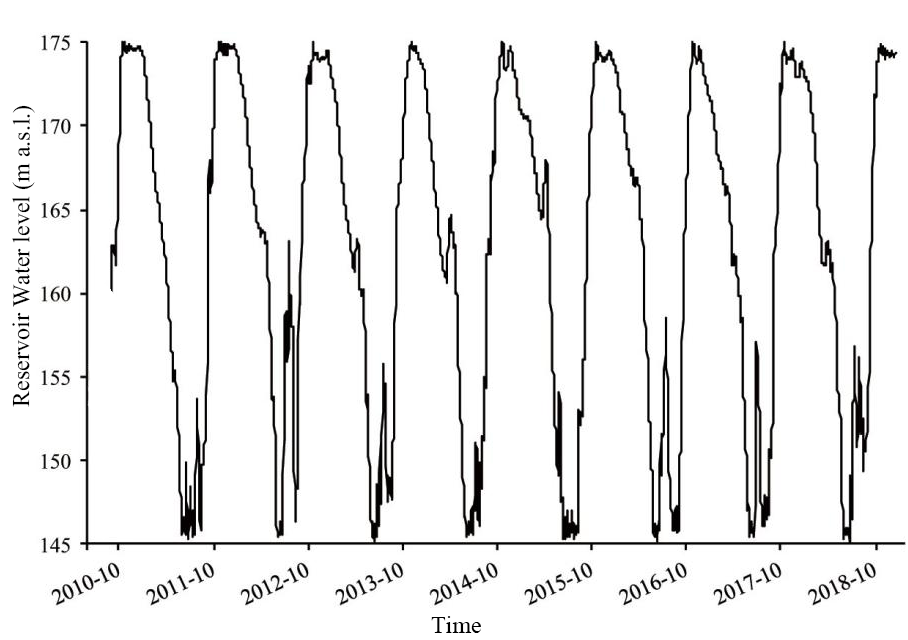


**Supplementary Figure 1.** The water-level changes in the Three Gorges Reservoir Region from October 2010 to October 2018. The shape of the annual hydrology is generally similar.

## 2. Supplementary Tables

**Supplementary Table 1.** The number of transects and quadrats and characteristics of submerged duration in different inundation gradients in the riparian zone of the Three Gorges Reservoir Region, China.

| Elevation zones | Flooding gradients | Submerged duration (d) | Number of transects | Number of quadrats |
| --- | --- | --- | --- | --- |
| 170-175 | Zone I | 68 | 62 | 186 |
| 165-170 | Zone II | 112 | 64 | 192 |
| 160-165 | Zone III | 152 | 98 | 294 |
| 145-160 | Zone IV | 204 | 103 | 309 |
| 145-175 | Overall | - | 327 | 981 |

**Supplementary Table 2.** Description of the selected plant functional traits used to measure functional diversity in the riparian zone of the Three Gorges Reservoir Region, China.

| Functional traits | Description | Measurement scale |
| --- | --- | --- |
| Dispersal type | Anemochory  Endozoochory & exozoochory  Ballistochory; myrmecochory; unassisted. | Nominal |
| Growth form | Graminoids  Herbs | Nominal |
| Life cycle | Annual  Biennial  Perennial | Nominal |
| Max. height (m) | 1: < 0.1 m  2: 0.11 – 0.29 m  3: 0.30 – 0.59 m  4: 0.60 – 0.99 m 5: 1.0 – 3.0 m | Ordinal |
| Flowering phenology | Spring flowering  Mid-summer flowering  Spring-mid-summer flowering  Fall flowering  Mid-summer-fall flowering | Nominal |

**Supplementary Table 3.** A list of plant species identified from the Three Gorges Reservoir Region, China.

| S. No. | Species | Genus | Family |
| --- | --- | --- | --- |
| 1 | *Abutilon theophrasti* | *Abutilon* | Malvaceae |
| 2 | *Acalypha australis* | *Acalypha* | Euphorbiaceae |
| 3 | *Euphorbia humifusa* | *Euphorbia* | Euphorbiaceae |
| 4 | *Euphorbia hypericifolia* | *Euphorbia* | Euphorbiaceae |
| 5 | *Euphorbia maculata* | *Euphorbia* | Euphorbiaceae |
| 6 | *Medicago lupulina* | *Medicago* | Fabaceae |
| 7 | *Medicago sativa* | *Medicago* | Fabaceae |
| 8 | *Kummerowia striata* | *Kummerowia* | Fabaceae |
| 9 | *Melilotus officinalis* | *Melilotus* | Fabaceae |
| 10 | *Trifolium repens* | *Trifolium* | Fabaceae |
| 11 | *Glycine soja* | *Glycine* | Fabaceae |
| 12 | *Aeschynomene indica* | *Aeschynomene* | Fabaceae |
| 13 | *Vicia tetrasperma* | *Vicia* | Fabaceae |
| 14 | *Vicia sativa* | *Vicia* | Fabaceae |
| 15 | *Vicia sepium* | *Vicia* | Fabaceae |
| 16 | *Dichrocephala auriculata* | *Dichrocephala* | Asteraceae |
| 17 | *Bidens tripartita* | *Bidens* | Asteraceae |
| 18 | *Xanthium sibiricum* | *Xanthium* | Asteraceae |
| 19 | *Kalimeris indica* | *Kalimeris* | Asteraceae |
| 20 | *Kalimeris lautureana* | *Kalimeris* | Asteraceae |
| 21 | *Dichrocephala benthamii* | *Dichrocephala* | Asteraceae |
| 22 | *Bidens pilosa* | *Bidens* | Asteraceae |
| 23 | *Ambrosia artemisiifolia* | *Ambrosia* | Asteraceae |
| 24 | *Artemisia argyi* | *Artemisia* | Asteraceae |
| 25 | *Ageratum conyzoides* | *Ageratum* | Asteraceae |
| 26 | *Cotula anthemoides* | *Cotula* | Asteraceae |
| 27 | *Artemisia annua* | *Artemisia* | Asteraceae |
| 28 | *Sonchus asper* | *Sonchus* | Asteraceae |
| 29 | *Senecio scandens* | *Senecio* | Asteraceae |
| 30 | *Artemisia selengensis* | *Artemisia* | Asteraceae |
| 31 | *Centipeda minima* | *Centipeda* | Asteraceae |
| 32 | *Eclipta prostrata* | *Eclipta* | Asteraceae |
| 33 | *Youngia japonica* | *Youngia* | Asteraceae |
| 34 | *Conyza canadensis* | *Conyza* | Asteraceae |
| 35 | *Gnaphalium affine* | *Gnaphalium* | Asteraceae |
| 36 | *Eupatorium coelestinum* | *Eupatorium* | Asteraceae |
| 37 | *Artemisia lavandulaefolia* | *Artemisia* | Asteraceae |
| 38 | *Soliva anthemifolia* | *Soliva* | Asteraceae |
| 39 | *Aster subulatus* | *Aster* | Asteraceae |
| 40 | *Conyza bonariensis* | *Conyza* | Asteraceae |
| 41 | *Crassocephalum crepidioides* | *Crassocephalum* | Asteraceae |
| 42 | *Amaranthus retroflexus* | *Amaranthus* | Amaranthaceae |
| 43 | *Chenopodium ambrosioides* | *Chenopodium* | Amaranthaceae |
| 44 | *Amaranthus spinosus* | *Amaranthus* | Amaranthaceae |
| 45 | *Amaranthus cruentus* | *Amaranthus* | Amaranthaceae |
| 46 | *Alternanthera sessilis* | *Alternanthera* | Amaranthaceae |
| 47 | *Alternanthera philoxeroides* | *Alternanthera* | Amaranthaceae |
| 48 | *Celosia argentea* | *Celosia* | Amaranthaceae |
| 49 | *Chenopodium album* | *Chenopodium* | Amaranthaceae |
| 50 | *Ampelopsis bodinieri* | *Ampelopsis* | Vitaceae |
| 51 | *Vitis heyneana* | *Vitis* | Vitaceae |
| 52 | *Vitis flexuosa* | *Vitis* | Vitaceae |
| 53 | *Centella asiatica* | *Centella* | Apiaceae |
| 54 | *Torilis japonica* | *Torilis* | Apiaceae |
| 55 | *Apium leptophyllum* | *Apium* | Apiaceae |
| 56 | *Oenanthe javanica* | *Oenanthe* | Apiaceae |
| 57 | *Daucus carota* | *Daucus* | Apiaceae |
| 58 | *Cryptotaenia japonica* | *Cryptotaenia* | Apiaceae |
| 59 | *Rorippa globosa* | *Rorippa* | Brassicaceae |
| 60 | *Rorippa indica* | *Rorippa* | Brassicaceae |
| 61 | *Capsella bursapastoris* | *Capsella* | Brassicaceae |
| 62 | *Arabidopsis thaliana* | *Arabidopsis* | Brassicaceae |
| 63 | *Elymus dahuricus* | *Elymus* | Poaceae |
| 64 | *Phragmites australis* | *Phragmites* | Poaceae |
| 65 | *Eleusine indica* | *Eleusine* | Poaceae |
| 66 | *Echinochloa crusgalli* var*.zelayensis* | *Echinochloa* | Poaceae |
| 67 | *Digitaria sanguinalis* | *Digitaria* | Poaceae |
| 68 | *Beckmannia syzigachne* | *Beckmannia* | Poaceae |
| 69 | *Eriochloa villosa* | *Eriochloa* | Poaceae |
| 70 | *Oplismenus compositus* | *Oplismenus* | Poaceae |
| 71 | *Arthraxon hispidus* | *Arthraxon* | Poaceae |
| 72 | *Cynodon dactylon* | *Cynodon* | Poaceae |
| 73 | *Echinochloa crusgalli var.austrojaponensis* | *Echinochloa* | Poaceae |
| 74 | *Echinochloa crusgalli* var*.mitis* | *Echinochloa* | Poaceae |
| 75 | *Leptochloa chinensis* | *Leptochloa* | Poaceae |
| 76 | *Setaria viridis* | *Setaria* | Poaceae |
| 77 | *Hemarthria altissima* | *Hemarthria* | Poaceae |
| 78 | *Paspalum paspaloides* | *Paspalum* | Poaceae |
| 79 | *Paspalum thunbergii* | *Paspalum* | Poaceae |
| 80 | *Echinochloa crusgalli* | *Echinochloa* | Poaceae |
| 81 | *Phalaris arundinacea* | *Phalaris* | Poaceae |
| 82 | *Echinochloa caudata* | *Echinochloa* | Poaceae |
| 83 | *Imperata cylindrica* | *Imperata* | Poaceae |
| 84 | *Buddleja davidii* | *Buddleja* | Scrophulariaceae |
| 85 | *Buddleja lindleyana* | *Buddleja* | Scrophulariaceae |
| 86 | *Calystegia hederacea* | *Calystegia* | Convolvulaceae |
| 87 | *Cuscuta japonica* | *Cuscuta* | Convolvulaceae |
| 88 | *Cuscuta chinensis* | *Cuscuta* | Convolvulaceae |
| 89 | *Pharbitis nil* | *Pharbitis* | Convolvulaceae |
| 90 | *Cardiospermum halicacabum* | *Cardiospermum* | Sapindaceae |
| 91 | *Mosla dianthera* | *Mosla* | Lamiaceae |
| 92 | *Leonurus sibiricus* | *Leonurus* | Lamiaceae |
| 93 | *Scutellaria franchetiana* | *Scutellaria* | Lamiaceae |
| 94 | *Clinopodium chinense* | *Clinopodium* | Lamiaceae |
| 95 | *Lagopsis supina* | *Lagopsis* | Lamiaceae |
| 96 | *Schizonepeta tenuifolia* | *Schizonepeta* | Lamiaceae |
| 97 | *Mosla scabra* | *Mosla* | Lamiaceae |
| 98 | *Stachys oblongifolia* | *Stachys* | Lamiaceae |
| 99 | *Salvia plebeia* | *Salvia* | Lamiaceae |
| 100 | *Perilla frutescens* | *Perilla* | Lamiaceae |
| 101 | *Commelina communis* | *Commelina* | Commelinaceae |
| 102 | *Commelina bengalensis* | *Commelina* | Commelinaceae |
| 103 | *Commelina diffusa* | *Commelina* | Commelinaceae |
| 104 | *Cyclosorus acuminatus* | *Cyclosorus* | Thelypteridaceae |
| 105 | *Cyperus rotundus* | *Cyperus* | Cyperaceae |
| 106 | *Cyperus michelianus* | *Cyperus* | Cyperaceae |
| 107 | *Fimbristylis dichotoma* | *Fimbristylis* | Cyperaceae |
| 108 | *Cyperus difformis* | *Cyperus* | Cyperaceae |
| 109 | *Cyperus iria* | *Cyperus* | Cyperaceae |
| 110 | *Solanum nigrum* | *Solanum* | Solanaceae |
| 111 | *Nicandra physalodes* | *Nicandra* | Solanaceae |
| 112 | *Physalis minima* | *Physalis* | Solanaceae |
| 113 | *Physalis angulata* | *Physalis* | Solanaceae |
| 114 | *Physalis alkekengi* | *Physalis* | Solanaceae |
| 115 | *Physalis pubescens* | *Physalis* | Solanaceae |
| 116 | *Solanum torvum* | *Solanum* | Solanaceae |
| 117 | *Datura innoxia* | *Datura* | Solanaceae |
| 118 | *Dioscorea bulbifera* | *Dioscorea* | Dioscoreaceae |
| 119 | *Duchesnea indica* | *Duchesnea* | Rosaceae |
| 120 | *Equisetum arvense* | *Equisetum* | Equisetaceae |
| 121 | *Equisetum hyemale* | *Equisetum* | Equisetaceae |
| 122 | *Ficus tikoua* | *Ficus* | Moraceae |
| 123 | *Galium aparine* | *Galium* | Rubiaceae |
| 124 | *Geranium nepalense* | *Geranium* | Geraniaceae |
| 125 | *Phyllanthus ussuriensis* | *Phyllanthus* | Phyllanthaceae |
| 126 | *Glochidion puberum* | *Glochidion* | Phyllanthaceae |
| 127 | *Phyllanthus urinaria* | *Phyllanthus* | Phyllanthaceae |
| 128 | *Gonostegia hirta* | *Gonostegia* | Urticaceae |
| 129 | *Pouzolzia zeylanica* | *Pouzolzia* | Urticaceae |
| 130 | *Houttuynia cordata* | *Houttuynia* | Saururaceae |
| 131 | *Humulus scandens* | *Humulus* | Cannabaceae |
| 132 | *Hypericum monogynum* | *Hypericum* | Hypericaceae |
| 133 | *Juncus prismatocarpus* | *Juncus* | Juncaceae |
| 134 | *Lindernia crustacea* | *Lindernia* | Linderniaceae |
| 135 | *Lindernia procumbens* | *Lindernia* | Linderniaceae |
| 136 | *Lindernia antipoda* | *Lindernia* | Linderniaceae |
| 137 | *Ludwigia prostrata* | *Ludwigia* | Onagraceae |
| 138 | *Lygodium japonicum* | *Lygodium* | Lygodiaceae |
| 139 | *Lysimachia christinae* | *Lysimachia* | Primulaceae |
| 140 | *Mazus japonicus* | *Mazus* | Phrymaceae |
| 141 | *Oxalis corniculata* | *Oxalis* | Oxalidaceae |
| 142 | *Phyla nodiflora* | *Phyla* | Verbenaceae |
| 143 | *Plantago asiatica* | *Plantago* | Plantaginaceae |
| 144 | *Veronica undulata* | *Veronica* | Plantaginaceae |
| 145 | *Veronica peregrina* | *Veronica* | Plantaginaceae |
| 146 | *Polygonum plebeium* | *Polygonum* | Polygonaceae |
| 147 | *Polygonum perfoliatum* | *Polygonum* | Polygonaceae |
| 148 | *Rumex dentatus* | *Rumex* | Polygonaceae |
| 149 | *Rumex chalepensis* | *Rumex* | Polygonaceae |
| 150 | *Polygonum lapathifolium* | *Polygonum* | Polygonaceae |
| 151 | *Rumex trisetifer* | *Rumex* | Polygonaceae |
| 152 | *Rumex acetosa* | *Rumex* | Polygonaceae |
| 153 | *Polygonum japonicum* | *Polygonum* | Polygonaceae |
| 154 | *Polygonum longisetum* | *Polygonum* | Polygonaceae |
| 155 | *Polygonum sibiricum* | *Polygonum* | Polygonaceae |
| 156 | *Polygonum hydropiper* | *Polygonum* | Polygonaceae |
| 157 | *Polygonum chinense* | *Polygonum* | Polygonaceae |
| 158 | *Polygonum criopolitanum* | *Polygonum* | Polygonaceae |
| 159 | *Portulaca oleracea* | *Portulaca* | Portulacaceae |
| 160 | *Pteris vittata* | *Pteris* | Pteridaceae |
| 161 | *Ranunculus sieboldii* | *Ranunculus* | Ranunculaceae |
| 162 | *Thalictrum simplex* | *Thalictrum* | Ranunculaceae |
| 163 | *Ranunculus sceleratus* | *Ranunculus* | Ranunculaceae |
| 164 | *Thalictrum aquilegifolium* | *Thalictrum* | Ranunculaceae |
| 165 | *Stellaria media* | *Stellaria* | Caryophyllaceae |
| 166 | *Trigonotis peduncularis* | *Trigonotis* | Boraginaceae |
